# Bioinformatics and Transcriptome Analysis of CFEM Proteins in *Fusarium graminearum*

**DOI:** 10.3390/jof7100871

**Published:** 2021-10-16

**Authors:** Lingqiao Chen, Haoyu Wang, Junhua Yang, Xianli Yang, Mengyuan Zhang, Zhihui Zhao, Yingying Fan, Cheng Wang, Jianhua Wang

**Affiliations:** 1 College of Food Science & Technology, Shanghai Ocean University, Shanghai 201306, China; m190300688@st.shou.edu.cn (L.C.); m200300781@st.shou.edu.cn (H.W.); m210300781@st.shou.edu.cn (M.Z.); 2 Shanghai Key Laboratory of Protected Horticultural Technology, Laboratory of Quality and Safety Risk Assessment for Agro-Products (Shanghai), Institute for Agro-Food Standards and Testing Technology, Ministry of Agriculture, Shanghai Academy of Agricultural Sciences, 1000 Jinqi Road, Shanghai 201403, China; yangjunhua@saas.sh.cn (J.Y.); yangxianli@saas.sh.cn (X.Y.); 18918162068@163.com (Z.Z.); 3 Institute of Quanlity Standards & Testing Technology for Agro-Products, Xinjiang Academy of Agricultural Sciences, Urumqi 830091, China; fyyxaas@xaas.ac.cn (Y.F.); wangcheng312@xaas.ac.cn (C.W.)

**Keywords:** *Fusarium graminearum*, CFEM domain, candidate effectors, bioinformatics analysis, transcriptional analysis

## Abstract

Fusarium blight of wheat is usually caused by *Fusarium graminearum*, and the pathogenic fungi will secrete effectors into the host plant tissue to affect its normal physiological process, so as to make it pathogenic. The CFEM (Common in Fungal Extracellular Membrane) protein domain is unique to fungi, but it is not found in all fungi. The CFEM protein contained in *F. graminearum* may be closely related to pathogenicity. In this study, 23 FgCFEM proteins were identified from the *F. graminearum* genome. Then, features of these proteins, such as signal peptide, subcellular localization, and transmembrane domains, etc., were analyzed and candidate effectors were screened out. Sequence alignment results revealed that each FgCFEM protein contains one CFEM domain. The amino acids of the CFEM domain are highly conserved and contain eight spaced cysteines, with the exception that FgCFEM8, 9, and 15 lack two cysteines and three cysteines were missed in FgCFEM18 and FgCFEM22. A recently identified CFEM_DR motif was detected in 11 FgCFEMs, and importantly we identified two new conserved motifs containing about 29 and 18 amino acids (CFEM_WR and CFEM_KF), respectively, in some of FgCFEM proteins. Transcriptome analysis of the genes encoding CFEM proteins indicated that all the CFEM-containing genes were expressed during wheat infection, with seven and six genes significantly up- and down-regulated, respectively, compared with in planta and in vitro. Based on the above analysis, FgCFEM11 and FgCFEM23 were predicted to be *F. graminearum* effectors. This study provides the basis for future functional analyses of CFEM proteins in *F. graminearum*.

## 1. Introduction

*Fusarium graminearum* species complex (FGSC) is an economically important plant pathogen causing Fusarium head blight (FHB) of wheat, barley, and other cereal crops worldwide [1,2]. FHB caused by FGSC is difficult to control and is known as the cancer of wheat. As one of the most destructive diseases worldwide, huge economic losses have been reported in Asia, Europe, North America, and many other countries [3]. FGSC has been ranked as the fourth most important plant pathogenic fungus [4]. In addition lowering grain yield, the disease mainly reduces grain quality, and results in mycotoxin-contaminated grain. Fusarium strains can produce epoxy-sesquiterpenoid compounds known as trichothecenes, with deoxynivalenol (DON) predominated. These secondary metabolites are very stable and can enter the food chain with food processing which pose potent threat to human and animal health, and thus has aroused public safety concerns. Some trichothecenes can also act as virulence factors for pathogenicity in susceptible plant hosts, which can facilitate colonization and spreading of the fungi in host tissues [5,6,7]. The prevalence and widespread outbreaks of the devastating FHB disease, exacerbated by recent changes in climate and certain cropping practices, has posed a threat for global wheat production and food safety [8].

According to the interaction patterns with the hosts, plant pathogens are classified into biotrophic, necrotrophic, and hemibiotrophic pathogens. The economically important and destructive hemibiotrophs, such as FGSC and *Magnaporthe oryzae*, were believed to be typical hemibiotrophic fungi [9] starting from the suppression of the host immune system in a biotrophic phase with living cells and followed by a later necrotrophic phase during which the pathogens kill plant cells for nutrient acquisition. However, the underlying interaction mechanisms between pathogens and plant hosts remain incompletely understood, especially in the early infection stages. The identification of proteinaceous effectors in a number of Ascomycetes, including biotrophic, necrotrophic, and hemibiotrophic pathogens is a typical example. Most fungal effector proteins identified so far are relatively small in size (usually <200 amino acids) and contain a high percentage (2% to 20%) of cysteine residues [10]. Thus, small secreted cysteine-rich proteins (SSCPs) are known to be a common source of fungal effectors that trigger resistance or susceptibility in specific host plants. Hence, identification and function characterization of SSCPs is of great importance for the understanding of the complex pathogenic process of the pathogens. 

Until now, several cysteine-rich domains have been identified in fungal proteins, such as those present in hydrophobins and epidermal growth factors (EGF) [11]. The CFEM (common in several fungal extracellular membrane proteins) domain is a unique motif found in fungi and usually sharing ~60 amino acids (AA), which contains eight conserved cysteine residues [12,13]. CFEM domain is found primarily in glycosylphosphatidylinositol (GPI)-anchored cell-wall proteins, and present in one or more copies (normally with one copy) near the N terminus of proteins. Although the domains in several hydrophobins also have eight cysteine residues, the domain size, cysteine spacing and pattern of residues is completely distinct when compared with those of CFEM [12,14,15]. CFEM is similar to several EGF-like domains in domain size and pattern of cysteine residues, however, the cysteine residues do not align with any of the EGF-like domains. The features of CFEM suggest that it is a novel domain with characteristics distinct from the known cysteine-rich domains. 

Many CFEM domain proteins have been found exclusively from Ascomycota and Basidiomycota, and they were found to be enriched in pathogenic fungi [13]. For example, there are 3, 8, 19, 10 CFEM proteins that have been identified in *Aspergillus fumigatus* [16], *Botryotinia fuckeliana* [13], *M. oryzae* [17], and *Sclerotinia sclerotiorum* [13], respectively. An average of 16 CFEM proteins were obtained from 12 released *Fusarium oxysporum* genomes by comparative analysis [18]. Most recently, nine CFEM effector candidates were identified from wheat leaf rust fungus *Puccinia triticina* [19]. Function studies indicated that CFEM-containing proteins involved in different functional categories [14,15,16,20,21,22]. In addition to their function as cell-surface receptors or signal transducers, or as adhesion molecules in host-pathogen interactions [12], some CFEM-containing proteins are proposed to play important roles in pathogenesis in some phytopathogenic fungi. PTH11 and ACI1 proteins produced by *Magnaporthe* *grisea* are required for appressorium development and subsequently plant infection [23,24]. Deletion of the CFEM domain of PTH11 led to defects in the differentiation of appressoria and appressoria-like structures in *M. oryzae* during the initiation of the blast disease in rice [17]. Target deletion of *BcCFEM1* in *Botrytis cinerea* suggested that the gene contributes to conidial production and stress tolerance, and most importantly the gene disrupt mutants resulted in decreased virulence on French bean (*Phaseolus vulgaris*) leaves, indicated that it was involved in pathogenicity [25]. Recently, the study by Arya et al. indicated that Bcin07g03260 (a non-GPCR membrane-bound CFEM protein) deletion mutants of *B. cinerea* also showed significantly reduced progression of a necrotic lesion on tomato (*Solanum lycopersicum*) leaves [26]. 

Many studies on identification and characterization of pathogenicity associated genes in *F. graminearum* have been reported. However, the underlying functions and mechanisms by which CFEM proteins act remain largely unknown in this pathogen. To our best knowledge, FGSG_03599 is the only *CFEM* gene that has been functionally characterized in the pathogen [27]. The status of systemic identification and function analysis of CFEM-containing proteins in *F. graminearum* are serious deficiencies. Considering the various functions of CFEM proteins in fungi, the objectives of the current study were to conduct a comprehensive bioinformatics analysis of CFEM proteins in *F. graminearum* based on the updated and re-annotated genome resource of PH-1 isolate [28].

## 2. Materials and Methods

### 2.1. Culture of Fungal Strain

The *F. graminearum* strain PH-1 (chemotype 15ADON, isolated on corn from Lansing, MI, USA) used throughout this study was routinely maintained on Potato Dextrose Agar (PDA) medium at 25 °C in the dark, unless otherwise specified. 

Fresh spores of PH-1 were generated in CMC (Per liter, the medium contained, 0.5 g NH_4_NO_3_, 0.5 g KH_2_PO_4_, 0.5 g MgSO_4_·7H_2_O, 0.5 g yeast extract, 7.5 g carboxymethyl cellulose-Na salt) medium with a 12:12 h light/dark cycle at 28 °C for 5 days with continuous shaking at 200 rpm. The conidial suspension was filtered through a two-layer Miracloth (Merck, Darmstadt, Germany) and centrifuged at 5000 rpm for 10 min. The harvested conidia were resuspended in sterile water and adjusted to 10^5^ conidia/mL with the aid of a hemacytometer. Sterile cotton strips were soaked in the conidial suspension for inoculation as described by Zhang et al. [29].

### 2.2. Plant Growth Conditions and Inoculation

The wheat cultivar Ningmai-13, a moderately resistant variety to FHB widely cultured in Jiangsu and Zhejiang, China, was used for the coleoptile infection assay according to Wu et al. [30] with modifications. Seeds were surface sterilized, washed, and germinated as described by Hao et al. [27]. Three days after seed sowing, the top 2–3 mm of the coleoptiles were removed, and the wounds were wrapped in cotton strips [29]. Mock inoculation using distilled water was carried out in parallel. After inoculation, the seedlings were grown in a growth chamber at 25 °C and 90% humidity. A scheme for the inoculation protocol can be found in Supplementary Figure S1. 

### 2.3. RNA Extraction and Microarray Hybridization

*F. graminearum*-inoculated coleoptiles were collected at 7 days post-inoculation (dpi). For each treatment, ten coleoptiles were collected and combined as one sample. Three independent biological replicates were performed for microarray assay. To prepare samples of mycelium grown in vitro for 7 d, cotton strips soaked the conidial suspension were transferred onto PDA plates with cellophane paper and cultivated at 25 °C for 7 d. Three replicates were included in control treatments. Harvested samples were immediately transferred into liquid nitrogen. RNA samples were collected from the *F. graminearum*-inoculated coleoptiles and mycelium grown on PDA plates was served as control in microarray analysis.

Total RNA was isolated using Trizol Reagent (Cat#15596-018, Life technologies, Carlsbad, CA, USA) according to the manufacturer’s instructions. The quality of RNA samples was assessed using an RNA 6000 Pico Assay Kit with a 2100 BioAnalyzer (Agilent Technologies, Santa Clara, CA, USA). Prior to labeling, qualified total RNA was purified by a RNeasy mini kit (Cat#74016, QIAGEN, Hilden, Germany) and a RNase-Free DNase Set (Cat#79254, QIAGEN, Hilden, Germany), according to the manufacturer’s guidelines. Chip hybridization, washes, and scanning were performed as described by Zhang et al. [31].

### 2.4. Bioinformatic Analysis of Common in Fungal Extracellular Membrane (CFEM)-Containing Proteins in F. Graminearum

#### 2.4.1. Identification of CFEM-Containing Proteins in *F. Graminearum* Genome

To identify the CFEM-containing proteins in the *F. graminearum* genome (hereafter mentioned as FgCFEM), the previously reported CFEM-containing protein FGSG_03599 was used as query in EnsemblFungi database (http://fungi.ensembl.org/index.html, accessed on 20 September 2021). All obtained proteins were further examined for the presence of the CFEM domain using the PFAM tool in the SMART website (http://smart.embl-heidelberg.de/, accessed on 20 September 2021). The set of identified FgCFEM domains was extracted and used to BLAST the *F. graminearum* proteins again to find all related sequences. 

To examine the distribution of *FgCFEM* gene features on *F. graminearum* chromosomes, we mapped the 23 *FgCFEM* genes on chromosomes. Chromosome location images were generated using Mapchart software V2.32 [32] to localize putative CFEM proteins of *F. graminearum*. 

#### 2.4.2. Signal Peptide, Transmembrane Domain, and Subcellular Localization Prediction

The SignalP 5.0 Server and TargetP 2.0 Server (https://services.healthtech.dtu.dk/, accessed on 20 September 2021) were used to analyze and predict N-terminal signal peptides (SP) and subcellular localization for predicting protein amino acid sequences, respectively. Additionally, transmembrane regions of CFEM proteins were predicted based on TMHMM (http://www.cbs.dtu.dk/services/TMHMM/, accessed on 20 September 2021). Subcellular localization prediction of CFEM-containing proteins was performed with both TargetP 2.0 Server (https://services.healthtech.dtu.dk/service.php?TargetP-2.0, accessed on 20 September 2021) and Wolf Psort (https://wolfpsort.hgc.jp/, accessed on 20 September 2021).

#### 2.4.3. Phylogenetic Analysis and Multiple Sequences Alignment

The AA sequences of CFEM, CFEM_DR domains, and the matured proteins (without SP) were used to create multiple protein sequence alignments using ClustalW using default settings. The neighbor-joining method was used to construct the phylogenetic tree based on AA sequence of domains using MEGA 5.0 [33]. The reliability of the nodes of the tree was evaluated by non-parametric bootstrapping using 1000 pseudo-replicates. 

#### 2.4.4. Domain Analysis of FgCFEM Proteins

The CFEM domain is a unique domain in fungi and may affect fungal infection and developmental processes. Recently, a new conserved motif, CFEM_DR, was identified by Ling et al. [18] in some CFEM-containing proteins of *F. oxysporum*. The expression results suggested that some CFEM_DR proteins might be associated with pathogenicity. Therefore, in this study we conducted the motif analysis, including but not limited to CFEM_DR motif, for all the identified CFEM proteins in *F. graminearum*. For domain detection, the MEME 4.0 software (https://meme-suite.org/meme/tools/meme, accessed on 20 September 2021) was used [34].

#### 2.4.5. Glycosylphosphatidylinositol (GPI) Modification Site Prediction

The potential GPI modification sites were predicted using the “GPI Modification Site Prediction in Fungi” tool on the website (https://mendel.imp.ac.at/gpi/fungi_server.html, accessed on 20 September 2021) [35].

#### 2.4.6. Analysis of Candidate Effectors of CFEM-Containing Proteins

Based on previous studies by Brown et al. [36], Lu and Edwards [10], proteins containing signal peptides and protein sequences no more than 300 AA without predicted transmembrane regions were considered as potential effectors in this study. The effector probability for each of the secreted protein was further evaluated using EffectorP [37].

## 3. Results

### 3.1. Bioinformatics Identification of CFEM Proteins in F. Graminearum

We searched the *F. graminearum* proteome for CFEM-containing proteins on the basis of their similarity to known CFEM protein. The proteins retrieved in this search were used to BLAST the *F. graminearum* proteins again to find all related sequences. A total of 22 CFEM proteins (FgCFEM1–22) were found in the *F. graminearum* genome (Table 1). Based on SMART analysis, the presence of the CFEM domain in these proteins was further verified. 

**Figure 1:**
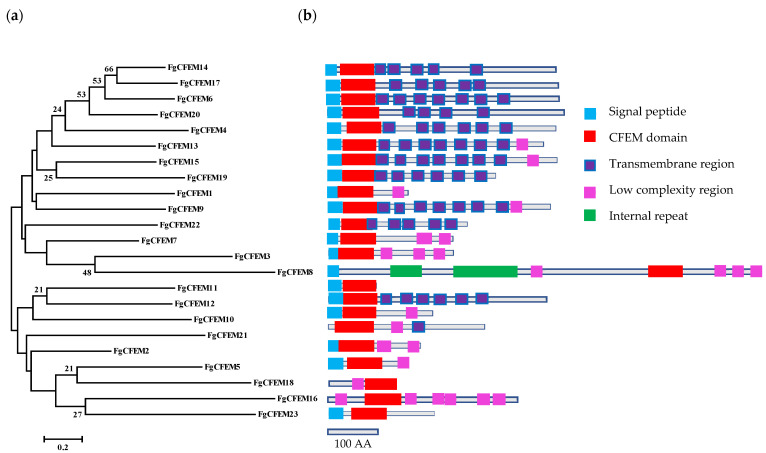
Phylogenetic analysis (**a**) and structure diagram (**b**) of the CFEM (Common in Fungal Extracellular Membrane) proteins in *F. graminearum.* A phylogenetic tree of adjacent connections was constructed based on amino acid sequences of FgCFEMs. The numbers on the nodes represent the percentage of their occurrences in the 1000 bootstrap replicates; the results show that more than 20% of the nodes are supported. The scale bar shows the number of amino acid differences at each site. The gray lines represent the length of each FgCFEM protein, the sky-blue box structure represents the signal peptide localization, red box represents CFEM structural domain, dark blue box represents transmembrane existence, purple represents low-degree complex regional protein, respectively. The scale represents the length of 100 AA in the architecture.

Previously, Zhang et al. [29] analyzed the expression of 21 *CFEM* genes in *F. graminearum* during the infection process in wheat. Of the genes analyzed in Zhang et al. [29] the homology of one *CFEM* gene, *FGSG_02840*, was not found in our BLAST analysis. According to SMART analysis, we confirmed that the protein encoded by *FGSG_02840* contains a CFEM domain, so the homology of this gene (new accession number FGRAMPH1_01G11435) was included in our study and named as *FgCFEM23*. Thus, it was predicted that there are 23 CFEM proteins encoded in the genome of *F. graminearum* genome (Table 1 and Figure 1). The different numbers of predicted CFEM proteins between the present research and previous studies may result from the newer genome database version. Among the 23 proteins, all were annotated as “hypothetical proteins” in the Ensembl Fungus Database. Only one CFEM domain was found in each FgCFEM proteins (Figure 1). 

**Figure 2:**
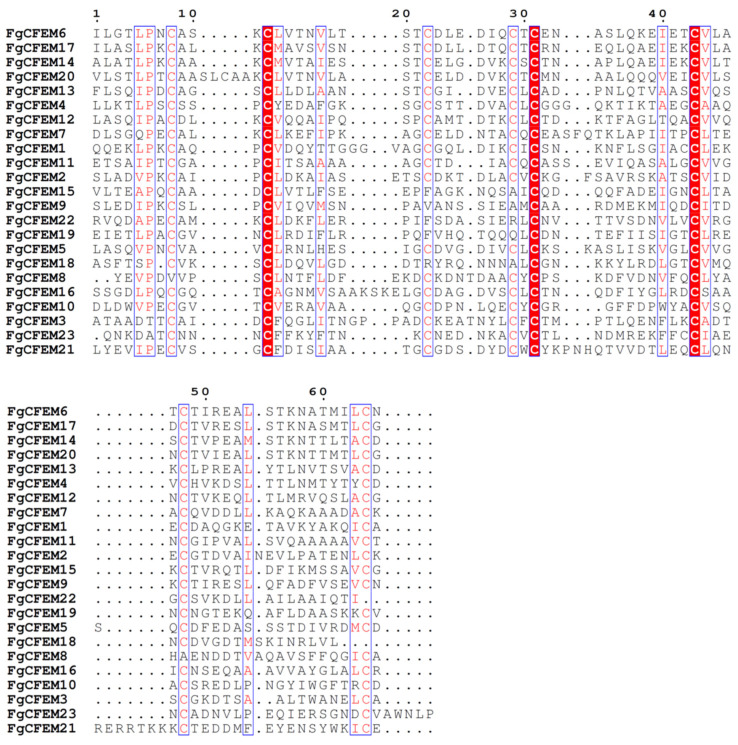
Multi-sequence alignment of the CFEM proteins in *F. graminearum.* The alignment was performed using ClustalW program, and the conserved amino acids are highlighted in red color. Red color font: conserved AA in some but not all the FgCFEMs, red color background: conserved AA in all the 23 FgCFEMs.

The full length (including SP) of these proteins ranged from 95 to 864 AA, of which FgCFEM11 is the shortest protein, accounting for 95 AA, and FgCFEM8 is the largest protein, accounting for 864 AA. On the other hand, the predicted mature proteins of the 23 CFEMs consisted of 77 to 847 AA with a majority (13 proteins) <400 AA. The smallest mature CFEM is FgCFEM11, which consisted of less than 100 AA. Cysteine residues in the mature proteins varied in number from 7 to 22 with a majority (18 proteins) ≤15, while the percentage of cysteine in mature proteins is 2.05% to 12.99% with most (18 proteins) <5%. Phylogenetic and multiple sequence alignment analysis revealed sequences conservation, and the eight cysteine residues in particular are well-conserved in most of these CFEM domains (Figure 2), which may be involved in the formation of disulfide bonds and play significant roles in the structure and function of the protein. 

### 3.2. Chromosomal Distribution of CFEM-Containing Genes

**Figure 3:**
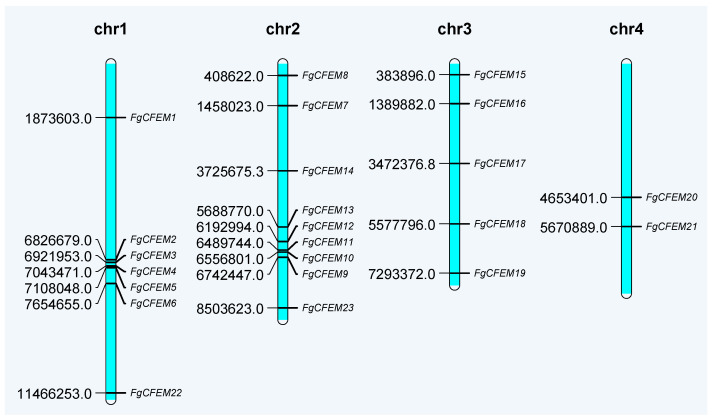
Mapping of the *CFEM* genes on *F. graminearum* (strain PH-1) chromosomes.

To determine the chromosomal distribution of putative CFEM-containing proteins, chromosome map was constructed from *F. graminearum* (Figure 3). The putative 23 *FgCFEM* genes are distributed among all four chromosomes, and chromosome 2 encoded the highest number, 9 genes of putative *FgCFEM* genes, followed by chromosome 1, 3, and 4, encoding 7, 5, and 2 genes, respectively. Overall, the genes encoding the 23 FgCFEMs appeared to be distributed randomly in the genome as they can be found in all four chromosomes with comparable numbers.

### 3.3. Feature Characterization of CFEM-Containing Proteins

We used the SignalP 5.0 Server to predict all CFEM protein signal peptides in *F. graminearum* and preliminarily determine the composition of these proteins. The results showed that 20 of the 23 CFEM proteins contained a signal peptide (Table 1 and Figure 1), whose sequence was a small segment of amino acid at the N-terminal. The SMART analysis result indicated that the initial amino acids between 15 to 23 encoded the signal peptides. No SP sequence was predicted in the remaining 3 FgCFEMs (FgCFEM16, 18, and 21) (Table 1). 

TargetP 2.0 and Wolf Psort analysis showed that 11 FgCFEM proteins were predicted as secretory proteins which could be secreted out of the cell through the secretion pathway of *F. graminearum* (Table 1).

According to TMHMM prediction, it demonstrated that 4–8 transmembrane regions were identified in 11 FgCFEMs and only one transmembrane region was found in FgCFEM21. No transmembrane region was found in the other 11 CFEM proteins (Table 1).

Further analysis of the 20 SP-containing FgCFEMs shows that 11 proteins have transmembrane domains and belonged to transmembrane proteins. The other nine CFEM proteins do not contain transmembrane domains and belong to secretory proteins. Moreover, of the 20 SP-containing proteins, nine were annotated by EnsemblFungi that consist of less than 300 AA in full-length (Table 1). 

GPI Modification Site Prediction (http://mendel.imp.ac.at/gpi/plant_server.html, accessed on 20 September 2021) was used to predict potential GPI modification site. The result demonstrated that two putative GPI modification sites were found in eight CFEMs, and the two identified amino acids are predicted to be the best and second best of potential GPI-modification sites, respectively (Table 1). For example, the amino acids N^139^ and G^131^, N^163^ and G^164^, S^220^ and A^228^ are the best and second best of potential GPI-modification sites in FgCFEM1, FgCFEM2, and FgCFEM3, respectively. No GPI modification site was predicted in the other 15 CFEMs. The results indicated that some *F. graminearum* CFEMs contain putative GPI-anchored sites, which are possibly anchored to the outer layer of the plasma membrane through an anchor or transferred to the cell wall as other GPI-anchored proteins in fungi [38]. According to previous studies, GPI-anchored proteins on the fungal cell wall have important effects on fungal adhesion, morphological transformation and cell wall synthesis, and microbial adhesion is one of the most important determinants of its pathogenicity. Therefore, to some extent, these CFEM proteins in *F. graminearum* are more likely to be associated with fungal disease.

### 3.4. Identification of Potential CFEM Effectors in F. Graminearum

Protein effectors are most often secreted via the conventional endoplasmic reticulum-Golgi apparatus rote, so normally they must contain an N-terminal secretion signal. Effector candidates can thus be identified bioinformatically by the presence of this signal [39]. Among the 23 proteins, 20 were found to contain an N-terminal signal peptide, which were preliminarily considered as the sources of secretory proteins. Combined with the subcellular localization and TMHMM analysis results, 11 FgCFEMs (FgCFEM1, 2, 3, 5, 7, 8, 10, 11,16, 18, 23) were selected as putative effectors based on the criteria that the predicted mature proteins belong to secretory proteins and do not contain transmembrane regions. 

All the 23 CFEMs were also evaluated using EffectorP [37] individually to predict their possibility as effectors. The results indicated that five proteins (FgCFEM1, 5, 11, 18, and 23) were predicted to be candidate effectors, which are all from the aforementioned 11 FgCFEMs. Our prediction result is consistent with the previous studies that FgCFEM11 (the homology of FGSG_03599), one of the CFEM proteins in *F. graminearum*, has been identified as an effector and functionally analyzed, which may be involved in plant infection [10,27]. Among the five candidate effectors, interestingly, FgCFEM18 is different from the other four CFEM proteins which contain no SP sequence by SignalP 5.0 prediction analysis. So probably FgCFEM18 is secreted through a novel pathway. Subsequently, the protein sequence of FgCFEM18 was submitted to the SecretomeP 2.0a Server (http://www.cbs.dtu.dk/services/SecretomeP/, accessed on 20 September 2021), and the result indicated that FgCFEM18 is a non-classically secreted protein with a NN-score 0.863 which means that the protein is probably secreted in non-classical pathways.

### 3.5. Phylogenetic Analysis of CFEM Proteins from F. Graminearum

**Figure 4:**
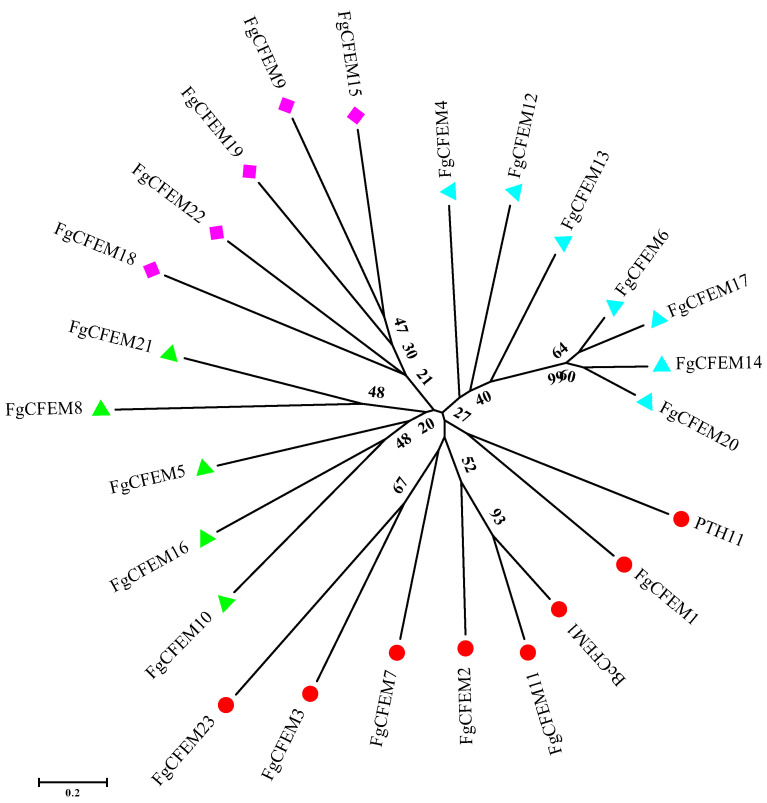
Phylogenetic analysis of the extracted CFEM domains of CFEM proteins in *F. graminearum*, PTH11 in *M. oryzae* and BcCFEM1 in *B. cinerea.* A phylogenetic tree of adjacent connections was constructed based on amino acid sequences of FgCFEMs. The numbers on the nodes represent the percentage of their occurrences in the 1000 bootstrap replicates; the results show that more than 20% of the nodes are supported. The scale bar shows the number of amino acid differences at each site. The different colors indicate different groups.

When compared at AA level, most CFEM proteins lacked significant similarities to each other. To elucidate the evolutionary relationships among the 23 CFEMs in *F. graminearum*, the sequences of CFEM domains were extracted for phylogenetic analysis. Moreover, two well-studied CFEM-containing proteins, PTH11 in *M. grisea* and BcCFEM1 in *B. cinerea*, respectively, were included to determine any relationships (Figure 4). 

According to the phylogenetic tree, all the 25 CFEMs can be divided into four major clades (Figure 4). Among them, FgCFEM14, 17, and 20 have relatively high homology, and the similarity is more than 52%. Additionally, the homology of FgCFEM11 and BcCFEM1 exceeded 42% and the two domains were clustered into a sub-group in phylogenetic analysis (Figure 4) while low CFEM domain identity (≤25%) was observed between PTH11 and all the FgCFEMs.

The structure of CFEM domains was also analyzed. The 23 CFEM domain sizes ranged from 61 to 75 AA with a majority (seven domains) at 65 AA. Among these CFEM domains, 17 domains contain the eight conserved cysteines which could form four disulfide bones to stabilize the whole protein structure [40]. However, two conserved cysteines were missed in the CFEM domains of FgCFEM8, 9, 15, and 19, and three cysteines were missed in FgCFEM18 and FgCFEM22 (Figure 2).

### 3.6. Conserved Motif Analysis

**Figure 5:**
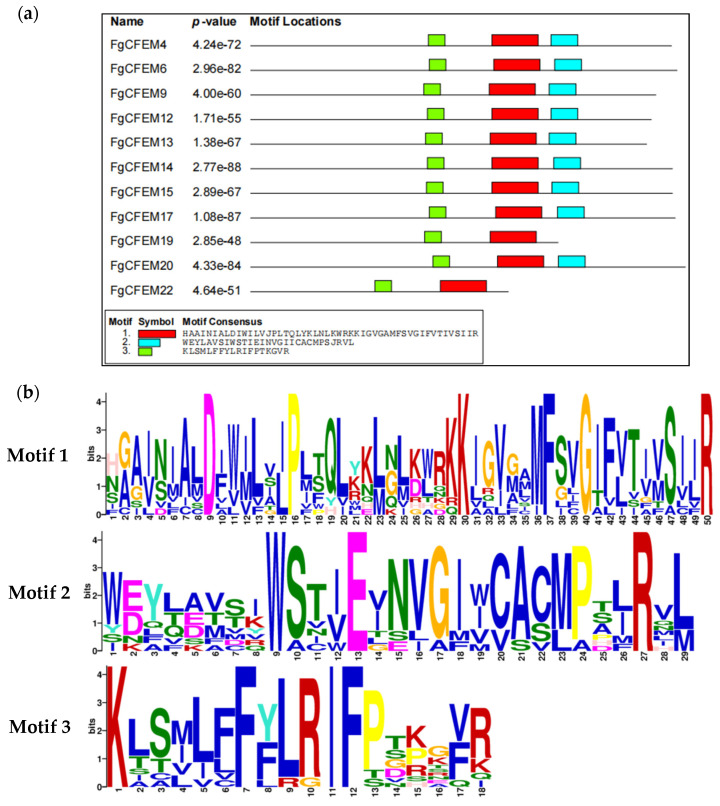
Identification of conserved motifs outside of CFEM domains by the MEME motif search tool. (**a**) Locations of new motifs identified. The black line represents the length of CFEM proteins, the red rectangle represents the motif 1, the blue rectangle represents motif 2, and the green protein represents motif 3; (**b**) a schematic diagram of motif 1, motif 2 and motif 3 and the conserved AA generated by MEME V4.0 software.

To reveal the potential conserved sequences of the CFEMs in *F. graminearum*, the MEME motif search tool was used to identify candidate motifs of these 23 proteins. By multiple sequence alignments of all 23 CFEM proteins, three blocks of conserved sequences outside the CFEM domain were detected in nine proteins (FgCFEM4, 6, 9, 12, 13, 14, 15, 17, 20) (Figure 5). Each block contained a conserved motif (Figure 5). For examples, motifs 1, 2, and 3 were conserved in xDxPxKxFxGxR, xWxExRx, and KxFxIFx patterns respectively. However, in FgCFEM19 and FgCFEM22 only motifs 1 and 3 were detected and no motif 2 was identified. For the other members, we failed to find any conserved motifs outside the CFEM domain, indicating the conserved cysteine amino acid sequence is the only feature for these proteins. Motif search results indicated that PHT11, the well studied CFEM protein in *M. oryzae*, contains all the three motifs, while none of these motifs were found in BcCFEM1 in *B. cinerea*. 

As shown in Figure 5, the conserved motif 1 resides on the C-terminal end of FgCFEM proteins and contains about 50 AA, six of which are conserved. The conserved residues of motif 1 detected in this study were consistent with previous work in which they were identified from *F. oxysporum* by Ling et al. [18], indicating that motif 1 belongs to CFEM_DR motif. Motif 2 and motif 3 are the novel conserved motifs. The conserved motif 3 resides in the middle of CFEM proteins and contains about 18 AA, four of which are conserved (KxFxIFx). The conserved motif 2 resides on the C-terminal end of CFEM proteins and contains about 29 AA, while only three conserved residues (xWxExRx) are detected. We referred to motif 2 and motif 3 as the WR motif and KF motif, respectively, in this study according to the first and last conserved amino acids in the motifs (WR and KF, respectively); the CFEM proteins containing the motif were referred as CFEM_WR and CFEM_KF proteins. Multiple sequence alignment of WR and KF motifs were given in Supplementary Figure S2. The identification of the novel motifs in some of the CFEM proteins indicated they might have divergent functions from the other CFEM proteins.

### 3.7. The Transcriptometrics Analysis of CFEM Genes in Planta

**Figure 6:**
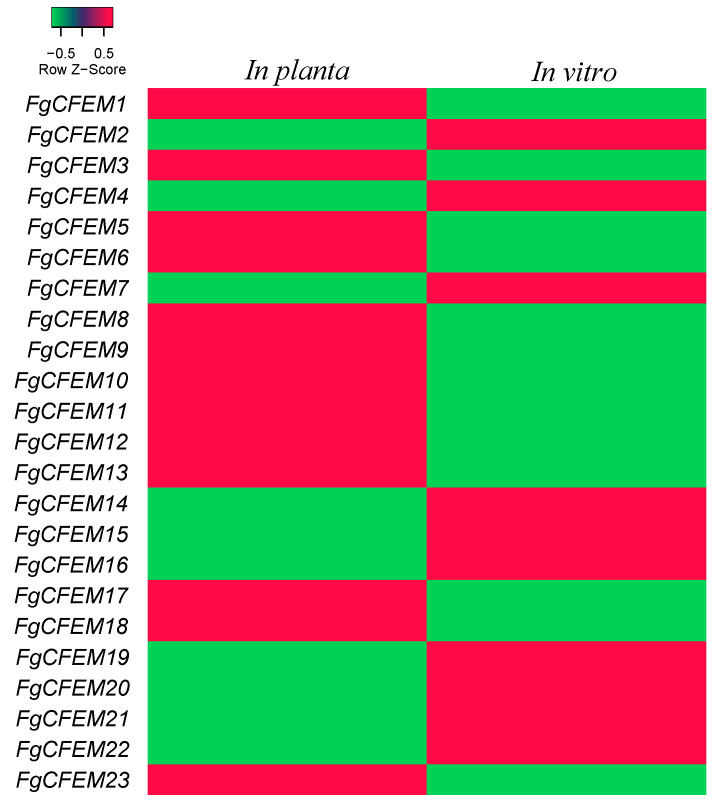
The heat map of *FgCFEM* genes. Green represents lower expression, red represents higher expression.

RNA derived from these samples was hybridized to the *F. graminearum* Affymetrix GeneChip. Transcriptional profiling of the CFEMs was carried out using a custom designed Agilent oligomer array, and the array contained up to three individual 60-mers for each gene. A total of 13,382 oligomers representing 13,382 fungal transcripts were perceived in the experiments. Microarray analyses revealed that the expression patterns of the 23 *CFEMs* differed greatly. The expression heatmap of these 23 *FgCFEM* genes are as shown in Figure 6. Specifically, the results of our microarray analysis showed that all the 23 *CFEM* genes were expressed at 7 dpi, with 7 genes (*FgCFEM6*, *8*, *11*, *12*, *13*, *17*, *23*) significantly up-regulated and six genes (*FgCFEM2*, *7*, *14*, *15*, *16*, *19*) significantly down-regulated in planta (fold change, FC ≥ 2). While no significant difference was found for the other 10 genes compared with in planta and in vitro. 

On the other hand, a genome-wide analysis of SSCPs was conducted by Lu and Edwards [10], and six CFEM-containing proteins (FgCFEM1, 2, 5, 7, 10, 11) were detected. Among the six genes, *FgCFEM1*, *2*, and *7* were constitutively expressed but no significant differences were observed compared with in planta and in vitro, whereas none of transcripts of *FgCFEM5* and *FgCFEM10* were detectable in planta infection or in vitro. Only the expression of *FgCFEM11* (the homology of *FGSG_03599*) was up regulated in comparison with those in fungal cultures. In our study, the expression pattern of *FgCFEM11* is consistent with the previous studies in Lu and Edwards [10]. 

Of the seven up-regulated *FgCFEM* genes identified in microarray analysis, only *FgCFEM11* and *FgCFEM23* were predicted to be effector candidates by bioinformatics analysis. The expression levels of the two genes in planta are 367.49 and 8.26 times higher, respectively, than in vitro. Both FgCFEM11 and FgCFEM23 contain a SP sequence at the N-terminal and no transmembrane region was identified for the two proteins. Considering the protein sizes, FgCFEM11 and FgCFEM23 accounting for 95 and 189 AA, respectively, indicating they are typical SSCPs. All the eight spaced cysteines were conserved in the two CFEM proteins by sequence alignment analysis. Combined with the transmembrane region prediction, subcellular localization analysis, EffectorP prediction and microarray results, we extrapolated that FgCFEM11 and FgCFEM 23 are the two effectors among the CFEM-containing proteins. 

## 4. Discussion

CFEM domain is unique to fungi and CFEM-containing proteins were found to be enriched in pathogenic fungi [13]. The roles of several CFEM proteins in different fungi were characterized. However, the specific roles of CFEM proteins in *F. graminearum* remain largely unknown. In the present study, we searched the proteome of *F. graminearum* for CFEM-containing proteins and identified a total of 23 sequences. This is the second largest number of CFEM candidates identified for any fungal species, and the largest number of this class of protein was identified in *Colletotrichum graminicola* [41]. However, only FGSG_03599, which is associated with virulence, has been reported [27]. All the identified CFEM-containing proteins in *F. graminearum* were annotated as hypothetical proteins in the new version of the strain PH-1 genome database. Sequence alignments revealed that most of the CFEM domains contain eight shared cysteine residues. The extreme amino-terminal and the carboxy-terminal sequences flanking this domain were divergent. This is consistent with other observations that sequences conservation is typically limited to the CFEM domains in CFEM-containing proteins. 

CFEM-containing proteins involved in different functional categories. CFEM-containing proteins have been found exclusively in fungi, especially in Ascomycota and Basidiomycota. For example, CFEM domain was found to participate in various functions mediating different physiological (cell wall stability [16,42]) and infection processes [25,43]. Studies of phytopathogenic fungi demonstrated the role of CFEM-containing proteins involved in different aspects of virulence. For example, the PTH11 and PTH11-like proteins are required for proper development of the appressoria, and pathogenicity in *M. oryzae* [17,43]. Similarly, in *M. grisea* [23], the adenylate cyclase (MAC1) CFEM-containing protein was shown to regulate appressorium formation. However, the underlying functions and mechanisms by which CFEM proteins act remain largely unknown in FGSC. Therefore, further research is both necessary and needed to answer these questions.

Among the 23 CFEM-containing proteins in *F. graminearum*, 20 were predicted to contain a SP and no SP sequence was detected in the other 3 proteins (FgCFEM16, 18, 21). These three proteins were further subjected to SecretomeP predictions for non-classically secrete proteins, and two of them (FgCFEM16, 21) obtain an NN-score exceeding the threshold (mammalian, 0.6) predicted by SecretomeP, indicating that these may be secreted in non-classical pathways. Non-classical secretory proteins were previously reported in secretome analysis of *F. graminearum*. Among the 69 unique fungal proteins identified, 11 were predicted to be secreted in a non-classical manner in *F. graminearum* [44]. Similarly, proteins secreted in a non-classical way in other fungi, e.g., *Aspergillus fumigates*, *Candida albicans*, *Claviceps purpurea*, and *Saccharomyces cerevisiae* have been reported previously [44,45].

The potential modification of CFEM-containing proteins was also analyzed in this study. The result demonstrated that putative GPI modification sites were found in eight CFEMs, indicating these may be GPI-anchored CFEM proteins. GPI-anchored proteins that can anchor to the outer layer of the plasma membrane through a C-terminal GPI anchor are essential for growth, signaling transmission, surface adhesion, and disease pathogenesis in eukaryotic cells [46]. For example, *BcCFEM1* encodes a CFEM protein with a putative GPI modification site in pathogen fungus *B. cinerea*. Disruption of this gene results in decreased virulence and increased sensitivity to osmotic and cell wall stress, indicating that BcCFEM1 is required for virulence and plays a key role in stress resistance [25]. The recent work by Arya et al. [26] illustrates a potential new role for a non-GPCR membrane CFEM in pathogenic fungi to control virulence in the fungus *B. cinerea*.

Effectors play critical roles during pathogen and plant interactions. Bioinformatics analysis predicted that there are about 600 effectors in the genome of *F. graminearum* [36]. Feature analysis indicated that many of these effectors are SCPPs that contain N-terminus signal peptides and lack transmembrane domains. In the study by Lu and Edwards [10], at least 34 SCPPs have been shown to be expressed in infected wheat heads. In this study, microarray analysis result indicated that all the CFEM-containing proteins were expressed during wheat infection, and seven genes were significantly up-regulated with *FgCFEM11* has the highest fold change in planta compared with in vitro. Based on the structure features and microarray analysis results, we conjectured that FgCFEM11 and FgCFEM23 are more probably to function as effectors during plant infection and may be involved in pathogenesis.

Conserved motif analysis indicated that 11 of the 23 FgCFEM proteins contained the CFEM_DR motif which recently identified in *F. oxysporum* [18]. In addition, by constructing phylogenetic tree and using MEME software, two new conserved motifs were identified by us in these 11 FgCFEM proteins. Future studies on the functional analysis of the two motifs should be conducted to investigate their potential roles during pathogen and plant interactions. 

## 5. Conclusions

In this study, we identified a total of 23 FgCFEM proteins in *F. graminearum* genome based on the recently updated and re-annotated genome resource of PH-1 isolate [28]. A comprehensive bioinformatics analysis on signal peptide, transmembrane domain, protein subcellular localization, and potential GPI modification sites of these CFEM proteins was conducted. The results showed that only 10 CFEM proteins were secreted proteins and GPI modification sites were identified in eight proteins. A subset of five effector candidates encoded by CFEMs was predicted using EffectorP [37]. Transcriptome analysis of all the 23 CFEM proteins was performed during infection process and seven CFEM-containing genes were highly expressed in wheat compared with control. Combined with the features characterization of CFEM proteins, we proposed that FgCFEM11 and FgCFEM23 are the two effectors among the CFEM-containing proteins in *F. graminearum*. Our findings provide a theoretical basis for in-depth analysis for the function of CFEM effectors in phytopathogenic fungi. 

## Figures and Tables

**Table 1:** The identification of Common in Fungal Extracellular Membrane (CFEM) proteins repertoire in *F. graminearum*.

Name	Protein ID	Amino Acid (AA)	No. of Cys	Cys% in Matured Protein	Position of CFEM Domain	SP Cleavage ^1^	mTP ^2^	SP	Other ^3^	Loc	TM ^4^ no.	GPI-Anchored	Effector
FgCFEM1	FGRAMPH1_01G01499	160	10	6.99%	18–85	17–18	0	0.9998	0.0002	S ^5^	0	N^139^/G^131^	Y ^7^
FgCFEM2	FGRAMPH1_01G05009	184	8	4.79%	19–86	17–18	0	0.9999	0.0001	S	0	N^163^/G^164^	-
FgCFEM3	FGRAMPH1_01G05085	250	9	3.90%	17–84	19–20	0	0.9998	0.0002	S	0	S^220^/A^228^	-
FgCFEM4	FGRAMPH1_01G05193	457	12	2.73%	25–90	17–18	0	0.9992	0.0008	- ^6^	6	-	-
FgCFEM5	FGRAMPH1_01G05255	161	8	5.80%	35–101	20–21	0.0005	0.9993	0.0002	S	0	N^138^/A^139^	Y
FgCFEM6	FGRAMPH1_01G05701	463	18	4.05%	28–92	19–20	0.0001	0.9925	0.0075	-	7	-	-
FgCFEM7	FGRAMPH1_01G10249	207	8	4.17%	17–84	15–16	0	0.9999	0.0001	S	0	G^176^/G^186^	-
FgCFEM8	FGRAMPH1_01G10975	864	22	2.60%	640–705	17–18	0	0.9996	0.0003	S	0	-	-
FgCFEM9	FGRAMPH1_01G13033	440	15	3.58%	23–87	21–22	0.0003	0.7624	0.2374	-	8	-	-
FgCFEM10	FGRAMPH1_01G13195	210	8	4.23%	27–90	21–22	0	0.9997	0.0003	S	0	S^186^/D^185^	-
FgCFEM11	FGRAMPH1_01G13253	95	10	12.99%	29–92	18–19	0	1	0	S	0	-	Y
FgCFEM12	FGRAMPH1_01G13513	435	17	4.10%	26–90	20–21	0	0.9991	0.0008	-	6	-	-
FgCFEM13	FGRAMPH1_01G13985	430	18	4.38%	25–88	19–20	0	0.9993	0.0007	-	7	-	-
FgCFEM14	FGRAMPH1_01G15521	458	15	3.42%	26–90	19–20	0.0004	0.9986	0.001	-	5	-	-
FgCFEM15	FGRAMPH1_01G16401	458	9	2.05%	26–90	20–21	0.0005	0.9338	0.0657	-	7	-	-
FgCFEM16	FGRAMPH1_01G17281	379	8	2.11%	73–142	−6	0.2608	0.0319	0.7073	S	0	N^355^/S^347^	-
FgCFEM17	FGRAMPH1_01G18835	461	15	3.40%	28–92	20–21	0.0009	0.9978	0.0013	-	5	-	-
FgCFEM18	FGRAMPH1_01G21361	129	7	5.43%	65–125	-	0.0007	0.4664	0.5329	S	0	-	Y
FgCFEM19	FGRAMPH1_01G21947	334	9	2.87%	24–88	20–21	0	0.9543	0.0457	-	6	-	-
FgCFEM20	FGRAMPH1_01G25789	472	18	3.97%	27–96	19–20	0.0001	0.9956	0.0043	-	4	-	-
FgCFEM21	FGRAMPH1_01G26539	312	8	2.56%	9–83	-	0	0	1	-	1	-	-
FgCFEM22	FGRAMPH1_01G08575	280	8	3.10%	26–88	22–23	0	0.906	0.094	-	5	-	-
FgCFEM23	FGRAMPH1_01G11435	189	10	5.92%	28–95	20–21	0	0.9994	0.0006	S	0	N^168^/G^169^	Y

^1^ SP cleavage, cleavage site of signal peptide (SP) in the FgCFEM proteins; ^2^ mTP, a mitochondrial targeting peptide prediction; ^3^ Other, any other localization; ^4^ TM, transmembrane domain; ^5^ S, secretory pathway; ^6^ -, no prediction; ^7^ Y, prediction is the effector.

## Data Availability

The data presented in this study are available on request from the corresponding author. The data are not publicly available because other data from these whole-genome transcriptomes are being used for other analyses to be published independently of this one.

## Supplementary Materials

The following are available online at https://www.mdpi.com/article/10.3390/jof7100871/s1, Figure S1: A scheme for wheat inoculation protocol, Figure S2: Multiple sequence alignment of WR and KF motifs.
