# Supplementary material for: Bioinformatics and Transcriptome Analysis of CFEM Proteins in Fusarium graminearum
Source: J Fungi (Basel). 2021 Oct 16;7(10):871. doi: 10.3390/jof7100871 (PMC8540330; doi:10.3390/jof7100871)
Supplement: Supplementary file 1 [file jof-07-00871-s001.zip › Figure S2.pdf]

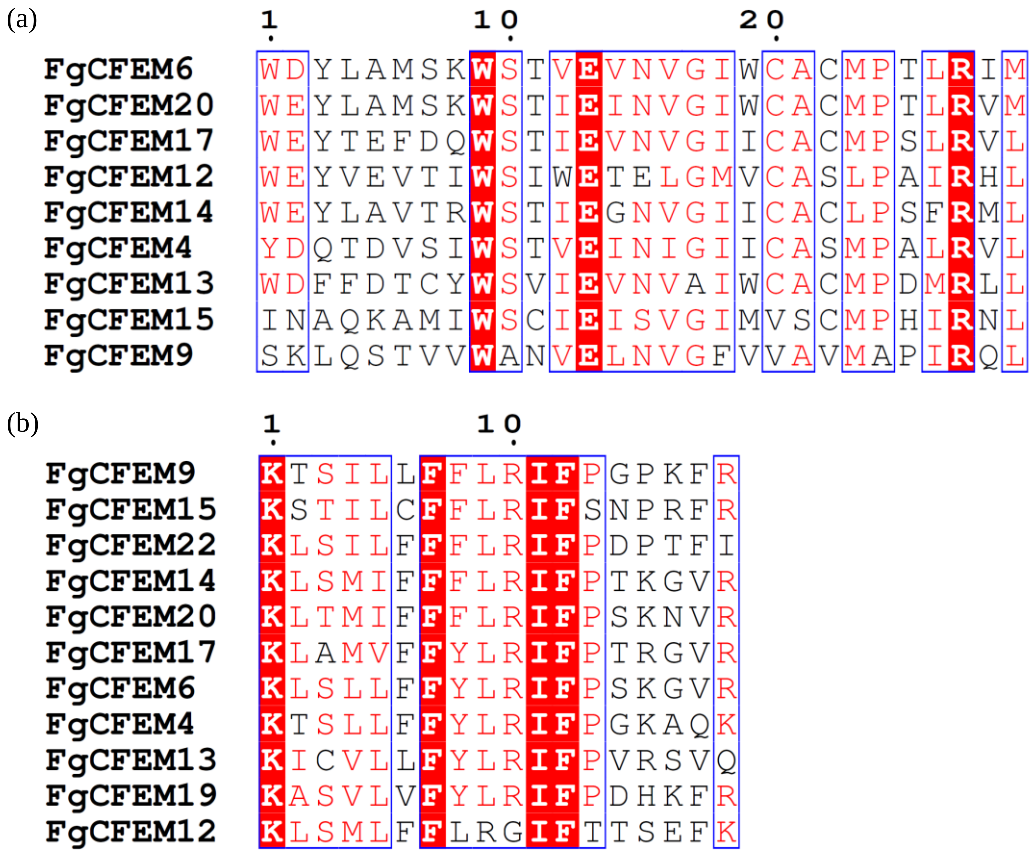

Figure S2. Multiple sequence alignment of WR (a) and KF (b) motifs. Red color font: conserved AA in some but not all the aligned FgCFEMs, red color background: conserved AA in all the aligned FgCFEMs.
