# Supplementary figures and images for: Bioinformatics and Transcriptome Analysis of CFEM Proteins in Fusarium graminearum
Source: J Fungi (Basel). 2021 Oct 16;7(10):871. doi: 10.3390/jof7100871 (PMC8540330; doi:10.3390/jof7100871)

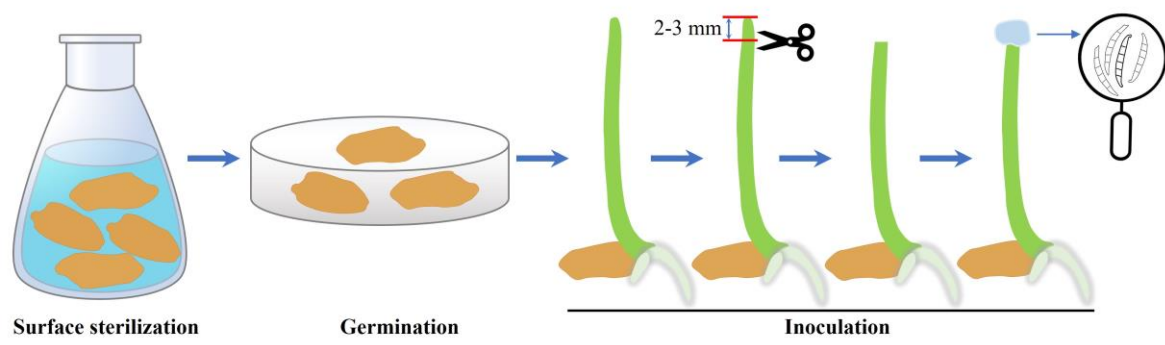

**Figure S1.** A scheme for wheat inoculation protocol.

Supplement: Supplementary file 1 [file jof-07-00871-s001.zip › Figure S1.pdf]
